# The contribution of the proximity of the retinal detachment to the fovea for postoperative metamorphopsia after 27-gauge pars plana vitrectomy for the primary rhegmatogenous retinal detachment

**DOI:** 10.1371/journal.pone.0258775

**Published:** 2021-10-28

**Authors:** Hiroko Yamada, Hisanori Imai, Akira Tetsumoto, Mayuka Hayashida, Keiko Otsuka, Akiko Miki, Makoto Nakamura

**Affiliations:** Department of Surgery, Division of Ophthalmology, Kobe University Graduate School of Medicine, Kobe, Japan; Medizinische Universitat Graz, AUSTRIA

## Abstract

**Purpose:**

To investigate clinical factors contributing to metamorphopsia after 27-gauge pars plana vitrectomy (27GPPV) for primary rhegmatogenous retinal detachment (RRD) to reveal whether the proximity of the preoperative retinal detachment to the fovea is associated with postoperative metamorphopsia.

**Methods:**

We retrospectively reviewed medical records of 77 eyes of 77 patients after 27GPPV for RRD. Patients were subdivided into three groups using optical coherence tomography findings: Group A, patients with RRD outside the vascular arcade; Group B, patients whose condition was present within the vascular arcade, but without foveal detachment; and Group C, patients with foveal detachment.

**Results:**

The average metamorphopsia score (°) assessed with M-charts 12 months after surgery was 0.01 ± 0.04 in Group A (24 eyes), 0.08 ± 0.18 in Group B (20 eyes), and 0.49 ± 0.48 in Group C (33 eyes) (p<0.001). Logistic regression analysis revealed that metamorphopsia at 12 months after surgery significantly correlated with the proximity of the retinal detachment to the fovea (p = 0.007).

**Conclusion:**

Metamorphopsia after 27GPPV for RRD correlated with the proximity of the preoperative retinal detachment to the fovea. Attention should be paid to the possibility of postoperative metamorphopsia development when retinal detachment is present within a vascular arcade even if the fovea is not involved.

## Introduction

In the past three years, the successful repair of primary rhegmatogenous retinal detachment (RRD) using microincision vitreous surgery, including 27-gauge (27G) pars plana vitrectomy (PPV), has been reported in 93–97.1% of cases [1–4]. Thus, PPV as a procedure performed for RRD has become particularly fine-tuned, yet it sometimes fails to yield sufficient improvement in the quality of vision despite successful structural repair of the RRD and a favorable postoperative best corrected visual acuity (BCVA). Such unsatisfactory results are due to disturbances in the vision such as metamorphopsia [5], aniseikonia [6], and impaired stereopsis [7].

Postoperative metamorphopsia reportedly develops in 20–49% of cases following vitreoretinal surgery for RRD [5, 8, 9] and is greatly affected by the preoperative condition of the retinal detachment and the integrity of the postoperative outer retinal structure [5, 8, 10, 11]. Specifically, it has been reported that postoperative metamorphopsia frequently occurs in the presence of preoperative foveal detachment, and its degree correlates with the abnormality in the postoperative outer retinal microstructure [5, 8, 10, 11]. Despite the absence of preoperative foveal detachment and the abnormality of the postoperative outer retinal microstructure, however, postoperative metamorphopsia occasionally develops, where the precise mechanism has been unknown [5, 10]. Therefore, we conducted a detailed examination of clinical factors associated with postoperative metamorphopsia after 27-gauge (27G) pars plana vitrectomy (PPV) to reveal whether the proximity of the preoperative retinal detachment to the fovea is associated with the occasional development of postoperative metamorphopsia, irrespective of the presence of preoperative foveal detachment and postoperative abnormalities of outer retinal microstructure.

## Material and methods

We retrospectively included 77 eyes of 77 consecutive patients who underwent uncomplicated 27GPPV for primary RRD and reviewed their medical records. This study was approved by the Ethics Committee of Kobe University Graduate School of Medicine (approval number: 180090) and it conformed to the tenets of the Declaration of Helsinki. Patients were enrolled from August 2016 to May 2019. In this study, informed consent was not obtained from each patient because of the retrospective, observational nature of the study. Although the need for informed consent was waived by ethics committee of Kobe University Graduate School of Medicine, patients were able to withdraw consent any-time for providing information about this study, which could be accessed on the hospital homepage as an opt-out choice. Eyes with giant tears, proliferative vitreoretinopathy (PVR), atopic dermatitis or a history of prior surgery for RRD were excluded. All patients were followed-up 12 months after the surgery. The enrolled patients were divided into three groups according to the macular status: Group A comprised patients whose RRD was located outside a vascular arcade; Group B comprised patients whose RRD was present within the vascular arcade, but did not involve the fovea, i.e., no foveal detachment; and Group C comprised patients with foveal detachment. The following variables were analyzed: age, sex, the lens status, axial length, period from subjective symptoms to surgery, location of retinal breaks, number of quadrants involved, the height of foveal detachment, presence or absence of foveal detachment, macular status, central macular thickness (CMT) 12 months after surgery, the continuity of EZ 12 months after surgery, presence or absence of ERM 3 and 12 months after surgery, preoperative BCVA, postoperative BCVA, postoperative metamorphopsia score, types of substances used as tamponade, operative time, success or failure of primary anatomical repair, success or failure of the final anatomical repair.

BCVA was measured before and 1, 3, 6, and 12 months after surgery. BCVA was converted to logarithmic minimum angle of resolution (log MAR) for statistical analysis. Metamorphopsia severity was quantified using M-CHARTS (Inami Co, Tokyo, Japan). M-CHARTS were recorded 1, 3, 6, and 12 months after the surgery. The horizontal, vertical, and average scores were used for the statistical analysis. In this study, a vertical or horizontal M-score of >0.2 was considered significant metamorphopsia.

**Fig 1:**
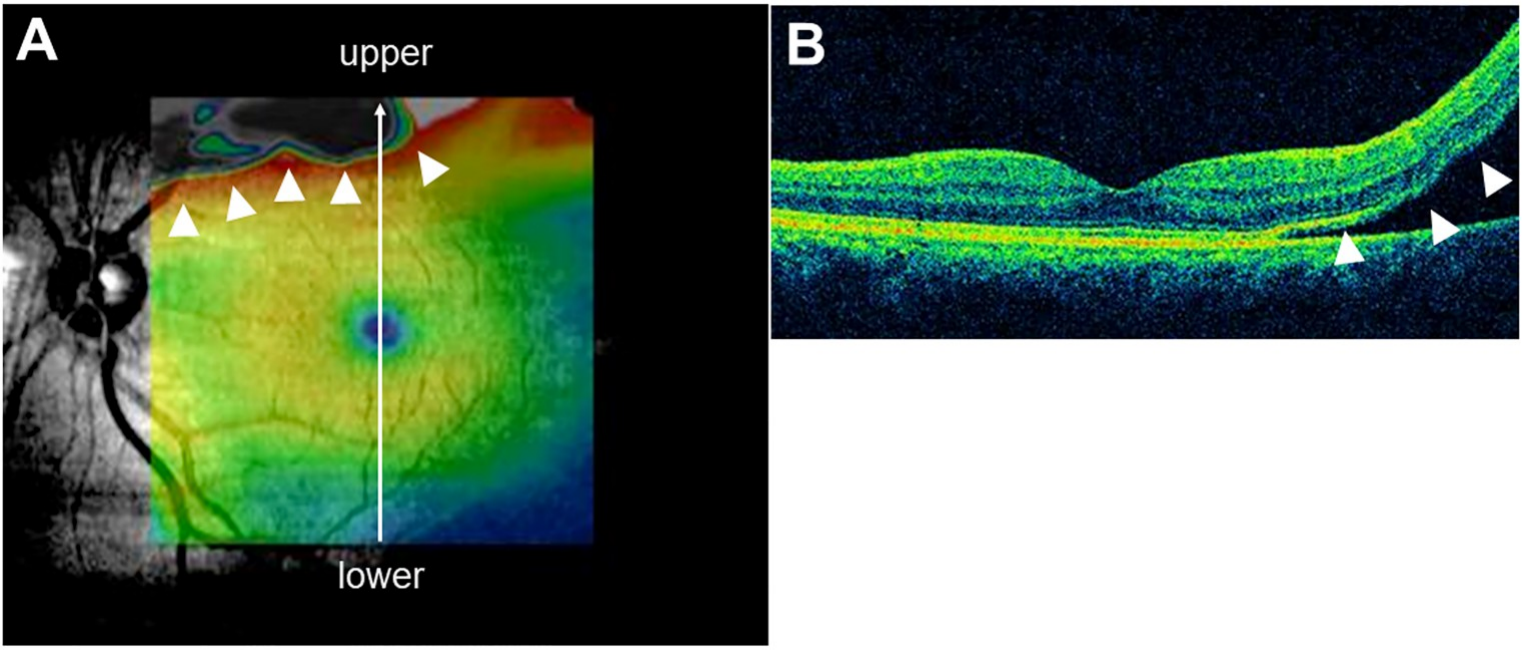
Images used to judge macular status. (A) A pseudocolor image overlaid on a fundus photograph. (B) Vertical cross-sectional B-scan image (white arrow in A) of an eye with RRD (white arrowheads in A, B) in the vascular arcade with no foveal detachment.

Spectral-domain OCT scans obtained using the Macular Cube 512 x 128 scan of the Cirrus HD-OCT (Cirrus HD-OCT model 4000, software Version 6.1.0.96; Carl Zeiss Meditec, Dublin, CA, USA) were used to judge the macular status (Fig 1), to categorize the height of foveal detachment, and to calculate the CMT. We assumed the range of pseudo color photographs of the Macular Cube 512 x 128 scan as an area of a vascular arcade. Eyes in which the pseudo color photograph did not capture the RRD, in which the RRD was captured by the pseudo color photograph but did not reach the fovea, and in which RRD reached the fovea, were classified as Group A, B, and C, respectively. By using the pseudo color photographs of the Macular Cube 512 x 128 scan, the distance from the retinal pigment epithelium (RPE) to the back of the exfoliated retina at the fovea was defined as height of foveal detachment. When the fovea was attached, it was categorized level A. All patients in Group A and Group B were categorized in Level A. In Group C, there are several cases where the both RPE and the back of the exfoliated retina cannot fit in one OCT photograph because the height of foveal detachment is too high. We categorized the RRD with foveal detachment that fits in one OCT photograph as Level B, and that did not fit in one OCT photograph as Level C. The B-scan images of a different spectral-domain OCT (Spectralis HRA + OCT; Heidelberg Engineering, Heidelberg, Germany) were used to evaluate the presence of the ERM and the continuity of the EZ. Volume scans (25 volume scans with 25 A-scans, 9.2 mm in length) and radial scans (6 radial scans with 30 A-scans each, 9.2 mm in length) were used for the evaluation.

The onset of subjective symptoms was defined as the onset of either sudden floaters, visual field loss, or subjective visual loss.

### Surgical procedures

All surgeries were performed at our clinic by 4 experienced vitreoretinal surgeons (H.I., A.M., W.M., and K.O.). Sub-Tenon anesthesia was administered using 4 ml of mixed 2% lidocaine and 0.5% levobupivacaine. The 27GPPV with ad wide-angle non-contact viewing system (Resight®; Carl Zeiss Meditec AG, Jena, Germany) was performed using the Constellation Vision System (Alcon Laboratories, Inc., Fort Worth, TX USA). Three cannulas were created with conjunctival displacement and oblique-angled sclerotomies in the inferotemporal, superotemporal, and superonasal quadrants 3.0–4.0 mm posterior to the limbus. A 27G chandelier illumination fiber (Oshima vivid, Synergetics, USA, Inc.) was placed 3.0–4.0 mm posterior to the limbus for wide-angle intraocular illumination. Before vitrectomy, phacoemulsification and intraocular lens implantation (PEA+IOL) with 2.4 mm bent transconjunctival single-plane sclera-corneal or clear corneal incision were performed using the same machine for all phakic eyes. Following core vitrectomy, the vitreous gel was visualized after injecting triamcinolone acetonide (MaQaid, Wakamoto Pharmaceutical, Tokyo, Japan) during midperipheral vitrectomy, and a complete 360° vitrectomy was done for the peripheral vitreous gel. Posterior vitreous detachment was present in all cases. Scleral indentation under a wide-angle non-contact viewing system was performed if necessary. No intentional drainage retinotomies were made. All retinal detachments were restored intraoperatively. Retinal photocoagulation was applied to completely surround all retinal breaks (Purepoint® endoocular probe, Alcon Laboratories, Inc., Fort Worth, TX, USA) (180 mW, 200 ms). At the end of the surgery, the eyes were flushed with 50 ml of 20% sulfur hexafluoride (SF6) or air, to ensure a complete exchange. An additional gas mixture was injected through the pars plana to adjust the intraocular pressure (IOP), if necessary. Any leaking sclerotomy site at the end of the surgery was closed with 8–0 vicryl suture. The IOP was checked by tactile examination. Subconjunctival corticosteroids were injected, and an antibiotic ointment was administered at the end of the surgical procedure. The scleral buckling procedure was not used on any case in this study. Perfluorocarbon liquid was not used either on any case in this study, because it cannot be used in Japan because its use for RRD without PVR is off-label. All patients were encouraged to adopt a prone position as soon as they moved from the operation bed to the wheelchair on the day of the surgery. All patients were encouraged to keep the designated position for at least 16 hours per day from the day after surgery to 1 week after the surgery. The designated position was decided depending on the location of the retinal breaks. Briefly, patients with temporal tears in the right eye were permitted lateral recumbent over the left side. Patients with nasal tears in the right eye were permitted lateral recumbent over the right side. Patients with inferior tears were permitted the lateral recumbent position on either side. Patients with superior tears were permitted to lay with the head upright. The designated position was not required during meals, bathroom visits, and sleeping hours, but the patients were encouraged to keep the designated positioning as much as possible. Adherence checks for postoperative postural restriction using monitors and frequent room visits were not performed.

### Statistical methods

For all variables, we reported the mean value and the standard deviation (SD). Friedman’s test and Wilcoxon t-test with Bonferroni correction for post hoc test were performed to assess changes in the visual functions with time (BCVA and M-score). Chi-square test and Fisher’s exact probability test for dichotomous variables and Mann-Whiney U-test and Kruskal Wallis test for continuous variables were used to compare the parameters between the groups. Logistic regression analysis was performed to determine the parameters associated with the presence of metamorphopsia 12 months after the surgery. Statistical analyses were performed using statistical software (SPSS, version 24.0; IBM Corporation, Armonk, NY, USA). Statistical significance was considered at p < 0.05.

## Results

Seventy-seven eyes of 77 patients were included in the study. The baseline demographic data were compared between the eyes with and without significant metamorphopsia and are summarized in Table 1. Significant metamorphopsia was present in 31 out of 77 patients 12 months after surgery. The preoperative BCVA (logMAR) was 0.39 ± 0.64 in all patients, 0.57 ± 0.54 in those with metamorphopsia, and 0.27 ± 0.68 in those without metamorphopsia (p<0.001). The BCVA 12 months after surgery was -0.08 ± 0.12, -0.04 ± 0.13, and -0.10 ± 0.10 in all patients, those with metamorphopsia, and those without metamorphopsia, respectively (p = 0.005). The two groups significantly differed in the axial length (p = 0.028), the height of foveal detachment (p<0.001), the presence or absence of preoperative foveal detachment (p<0.001), preoperative macular status (p<0.001) and ellipsoid zone (EZ) disruption 12 months after surgery (p<0.001). However, no statistical significance was found with respect to age, sex, lens status, period from subjective symptoms to surgery, location of retinal breaks, quadrant of retinal detachment, CMT 12 months after surgery, presence of epiretinal membrane (ERM) 3 and 12 months after surgery, types of tamponade substances, and the operative time.

**Table 1:** Comparison of the demographic data of patients with or without postoperative metamorphopsia.

	total	Metamorphopsia(+)	Metamorphopsia(-)	P Value
Number of Eyes	77	31	46	
Age (years), mean±SD	58.68±10.15	58.10±9.4	59.07±10.68	0.992
Sex, male/female	43/34	20/11	23/23	0.208
Lens status, phakia/pseudophakia/aphakia	67/10/0	26/5/0	41/5/0	0.743
Axial Length (mm), mean±SD	25.23±1.47	25.69±1.23	24.92±1.52	0.028*
Period from subjective symptoms to surgery (days)	7.13±11.22	4.87±6.36	8.61±13.34	0.242
Location of retinal breaks, superior/inferior/3 or 9 o’clock	46/19/12	17/9/5	29/10/7	0.734
Quadrant of retinal detachment, 1/2/3/4	22/49/5/1	5/23/2/1	17/26/3/0	0.058
Height of foveal detachment, level A/B/C	44/20/13	8/7/16	36/3/7	<0.001**
Foveal detachment, +/-	33/44	23/8	10/36	<0.001**
Macular status, Group A/B/C	24/20/33	2/6/23	22/14/10	<0.001**
EZ disruption 12 months after surgery, +/-	17/60	15/16	2/44	<0.001**
CMT 12 months after surgery(μm), mean±SD	281.66±33.02	275.23±33.24	286.00±32.51	0.133
Presence of ERM 3 months after surgery,+/-	10/67	4/27	6/40	0.743
Presence of ERM 12 months after surgery,+/-	14/63	7/24	7/39	0.318
Preoperative BCVA (logMAR), mean±SD	0.39±0.64	0.57±0.54	0.27±0.68	0.001*
BCVA (logMAR) 12 months after surgery, mean±SD	-0.08±0.12	-0.04±0.13	-0.10±0.10	0.005*
Tamponade substance, air/SF6gas	62/15	27/4	35/11	0.366
Operative time (minutes), mean±SD	83.79±36.38	89.55±45.36	79.91±28.73	0.708
Initial anatomical success, no. (%)	77(100)	31(100)	46(100)	
Final anatomical success, no. (%)	77(100)	31(100)	46(100)	

*Significant at P < 0.05

**Significant at P <0.001; SD, standard deviation; logMAR, logarithm of the minimal angle of resolution.

BCVA, best-corrected visual acuity; ERM, epiretinal membrane; EZ, ellipsoid zone; CMT, central macular thickness.

**Fig 2:**
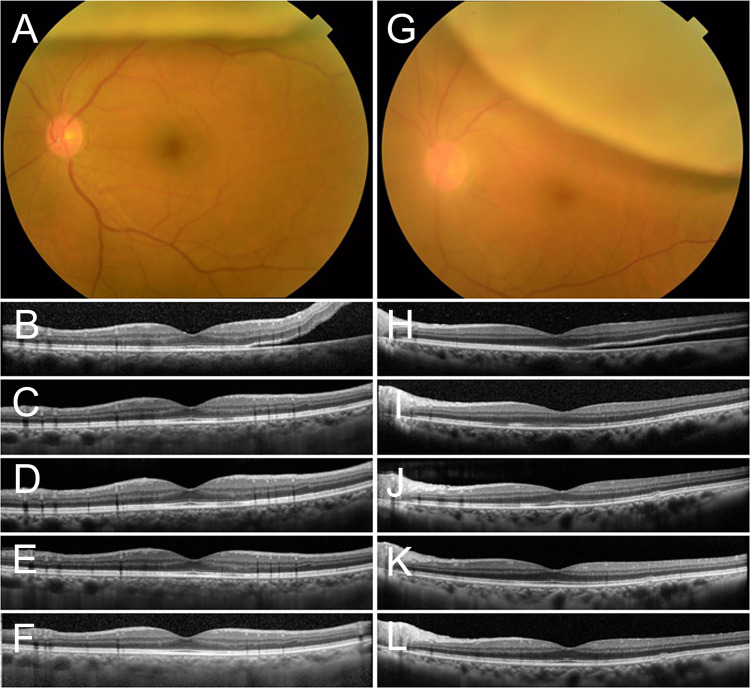
Time course of OCT findings in representative patients whose RRD were present within the vascular arcade but did not involve the fovea. Left panel: A, the preoperative fundus Images of the left eye of a 62-year-old man. The subfoveal OCT findings were normal during the follow-up period (B: Pre, C: 1 months, D: 3 months, E: 6 months, F: 12 months after the surgery, respectively). M-score was normal during the follow-up period. Right Panel: G, the preoperative fundus Images of the left eye of a 62-year-old man. The subfoveal OCT findings were normal during the follow-up period (H: Pre, I: 1 months, J: 3 months, K: 6 months, L: 12 months after the surgery, respectively). Preoperative M-score was normal, but vertical and horizontal M-score were 0.4 and 0.3 after 12months surgery, respectively.

Based on the results in Table 1, logistic regression analysis was performed. The presence or absence of metamorphopsia was used as the dependent variable. First, the presence or absence of foveal detachment, the axial length, height of foveal detachment, EZ disruption 12 months after surgery, preoperative BCVA (logMAR), and BCVA (logMAR) 12 months after surgery were included as independent variables. As shown in Table 2, the presence of preoperative foveal detachment (P = 0.002), height of foveal detachment (p = 0.046), and EZ disruption 12 months after surgery (p = 0.013) were significantly associated with the presence of postoperative metamorphopsia. Next, the preoperative macular status, the axial length, height of foveal detachment, EZ disruption 12 months after surgery, preoperative BCVA (logMAR), and BCVA (logMAR) 12 months after surgery were included as independent variables. As shown in Table 3, preoperative macular status (p = 0.007) and EZ disruption 12 months after surgery (p = 0.027) were significantly associated with the presence or absence of postoperative metamorphopsia, but other factors were not associated with the presence or absence of postoperative metamorphopsia. Fig 2 shows OCT findings in representative patients whose RRD were present within the vascular arcade, but did not involve the fovea.

**Table 2:** Logistic regression analysis including the presence or absence of preoperative foveal detachment as an independent variable.

Parameter	β	SE	P Value
Foveal detachment	5.929	1.936	0.002
Axial Length	-0.288	0.246	0.241
Height of foveal detachment	2.290	1.148	0.046
EZ disruption 12 months after surgery	2.437	0.983	0.013
Preoperative BCVA (logMAR)	0.838	0.940	0.373
BCVA (logMAR) 12 months after surgery	-2.104	3.403	0.536

*Significant at P< 0.05; **Significant at P <0.001; β, partial correlation coefficient; SE, standard error

EZ, ellipsoid zone; BCVA, best-corrected visual acuity; logMAR, logarithm of the minimal angle of resolution

**Table 3:** Logistic regression analysis including the preoperative macular status as an independent variable.

Parameter	β	SE	P Value
Macular status	-2.320	0.867	0.007
Axial Length	-0.153	0.234	0.514
Height of foveal detachment	0.762	0.826	0.356
EZ disruption 12 months after surgery	1.978	0.894	0.027
Preoperative BCVA (logMAR)	0.558	0.831	0.502
BCVA (logMAR) 12 months after surgery	-1.359	3.332	0.683

*Significant at P< 0.05; β, partial correlation coefficient; SE, standard error.

BCVA, best-corrected visual acuity; logMAR: logarithm of the minimal angle of resolution; EZ, ellipsoid zone.

Since the preoperative macular status contributed to the presence or absence of postoperative metamorphopsia, we compared all the parameters, the course change of metamorphopsia, and the course change of BCVA among the groups.

The demographic data compared among the groups are summarized in Table 4. There were 24 eyes in Group A, 20 eyes in Group B, and 33 eyes in Group C. The axial length (mm) was 24.57 ± 1.20 in Group A, 25.45 ± 1.75 in Group B, 25.64±1.30 in Group C (p = 0.020). The number of quadrants of RRD involved significantly differed among groups (p<0.001). About height of foveal detachment, all patients in Group A and Group B were categorized in Level A. In Group C, 19 patients were categorized in Level B, and 20 were in Level C (p<0.001). There were no eyes with EZ disruption in Group A, 3 (15%) of 20 eyes with EZ disruption in Group B and 14 (42.4%) of 33 eyes with EZ disruption in Group C (p<0.001). Preoperative BCVA (logMAR) was 0.01 ± 0.35 in Group A, -0.02 ± 0.14 in Group B, 0.92 ± 0.61 in Group C (p<0.001). BCVA at 12 months after surgery was -0.12 ± 0.08 in Group A, -0.11 ± 0.10 in Group B, -0.02 ± 0.13 in Group C (p<0.001). The average metamorphopsia score (°) 12 months after surgery was 0.24 ± 0.40, 0.01 ± 0.04, 0.08 ± 0.18, and 0.49 ± 0.48 in all patients, Group A, Group B, and Group C, respectively (p<0.001). Of the 24 patients in Group A, 20 patients in group B, and 33 patients in group C, 2 (2/24, 8.3%), 6 (6/20, 30%) and 23 (23/33, 69.7%), respectively had significant metamorphopsia (p<0.001).

**Table 4:** Comparison of the demographic data of patients among groups.

		total	Group A	Group B	Group C	P Value
Number of Eyes		77	24	20	33	
Age (years), mean±SD		58.68±10.15	60.38±11.17	56.35±9.28	58.85±9.91	0.505
Sex, male/female		43/34	10/14	11/9	22/11	0.175
Lens status, phakia/pseudophakia/aphakia		67/10/0	22/2/0	18/2/0	27/6/0	0.763
Axial Length (mm), mean±SD		25.23±1.47	24.57±1.20	25.45±1.75	25.64±1.30	0.020*
Period from subjective symptoms to surgery (days)		7.13±11.22	6.88±12.42	10.45±15.03	5.25±6.37	0.379
Location of retinal breaks, superior/inferior/3 or 9 o’clock		46/19/12	16/4/4	13/5/2	17/10/6	0.49
Quadrant of retinal detachment, 1/2/3/4		22/49/5/1	13/11/0/0	7/13/0/0	2/25/5/1	<0.001**
Height of foveal detachment, Level A/B/C		44/20/13	24/0/0	20/0/0	0/20/13	<0.001**
EZ disruption 12 months after surgery, +/-		17/60	0/24	3/17	14/19	<0.001**
CMT 12 months after surgery (μm), means±SD		281.66±33.12	288.25±27.21	282.95±34.25	276.09±35.99	0.129
Presence of ERM 3 months after surgery, +/-		10/67	3/21	5/15	2/31	0.284
Presence of ERM 12 months after surgery, +/-		14/63	4/20	5/15	5/28	0.649
Preoperative BCVA (logMAR), means		0.39±0.64	0.01±0.35	-0.02±0.14	0.92±0.61	<0.001**
BCVA (logMAR) 12 months after surgery, mean±SD		-0.08±0.12	-0.12 ± 0.08	-0.11 ± 0.10	-0.02 ± 0.13	<0.001**
Metamorphopsia score 12 months after surgery	Average (°)	0.24±0.40	0.01±0.04	0.08±0.18	0.49 ± 0.48	<0.001**
	Horizontal (°)	0.23±0.44	0.00±0.00	0.05±0.13	0.51±0.57	<0.001**
	Vertical (°)	0.23±0.38	0.02±0.07	0.12±0.25	0.45±0.47	<0.001**
Presence of metamorphopsia, +/-		31/46	2/22	6/14	23/10	<0.001**
Tamponade substance, air/SF6 gas		62/15	18/6	16/4	28/5	0.81
Operative time (minutes), mean±SD		83.79±36.38	79.29±30.65	87.80±33.76	84.64±42.02	0.587
Initial anatomical success, no. (%)		77(100)	24(100)	20(100)	33(100)	
Final anatomical success, no. (%)		77(100)	24(100)	20(100)	33(100)	

*Significant at P < 0.05

**Significant at P <0.001; SD, standard deviation; logMAR, logarithm of the minimal angle of resolution.

BCVA, best-corrected visual acuity; EZ, ellipsoid zone. ERM, epiretinal membrane; CMT, central macular thickness.

**Fig 3:**
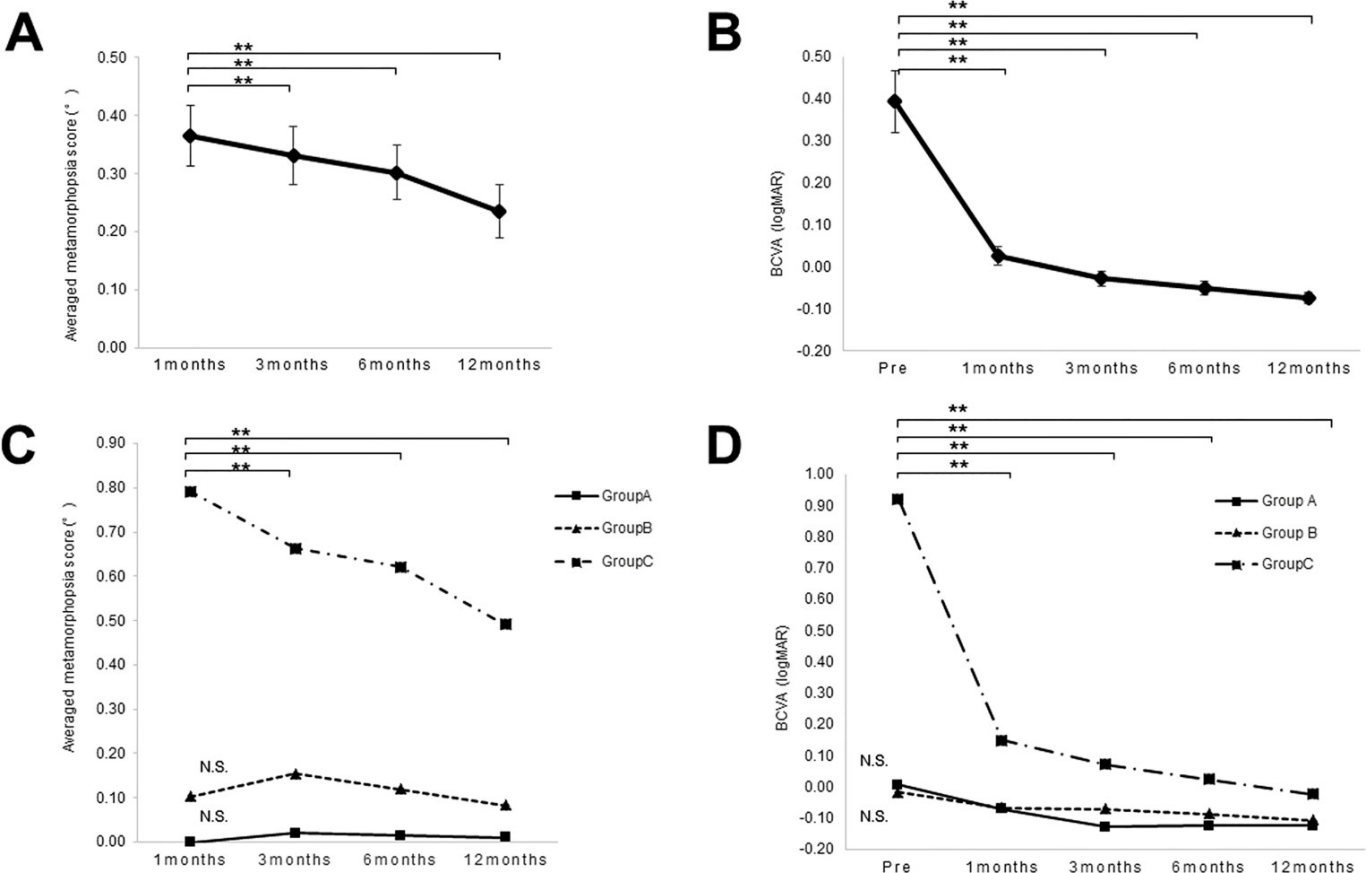
Changes in visual functions of patients after RRD surgery. In all cases, the average metamorphopsia score at 3, 6, and 12 months postoperatively significantly improved from the score at 1 month (p< 0.01) (A). The average metamorphopsia score at 3, 6, and 12 months postoperatively significantly improved from the score at 1 month in Group C (p< 0.01) (C). The average metamorphopsia score did not change throughout the follow-up period in Groups A and B (C). In all cases, BCVA (logMAR) at 1, 3, 6, and 12 months postoperatively significantly improved compared with preoperative BCVA (P < 0.01) (B). BCVA (logMAR) at 1, 3, 6, and 12 months postoperatively significantly improved compared with preoperative BCVA in Group C (P < 0.01) (D). BCVA (logMAR) did not change throughout the follow-up period in Groups A and B (D).

Fig 3 shows changes in the visual functions of patients after surgery for RRD. In all cases, the average metamorphopsia score at 1, 3, 6 and 12 months postoperatively were 0.37 ± 0.46, 0.33 ± 0.44, 0.30 ± 0.41, and 0.24 ± 0.40, respectively (p = 0.008). The average metamorphopsia score at 3, 6, and 12 months postoperatively significantly improved from the score at 1 month (p< 0.01) (Fig 3A). In Group A, the average metamorphopsia scores at 1, 3, 6, and 12 months postoperatively were 0.00 ± 0.00, 0.02 ± 0.06, 0.01 ± 0.04, and 0.01 ± 0.04, respectively (p = 0.896); In Group B, they were 0.10 ± 0.15, 0.16 ± 0.16, 0.12 ± 0.19, and 0.08 ± 0.18, respectively (p = 0.641); and in Group C, they were 0.79 ± 0.41, 0.66 ± 0.45, 0.62 ± 0.44, and 0.49 ± 0.48, respectively (p<0.01). The average metamorphopsia scores at 3, 6, and 12 months postoperatively significantly improved from the score at 1 month in Group C (p< 0.01) (Fig 3C), but did not change throughout the follow-up period in Groups A and B (Fig 3C). For all patients, the preoperative BCVA (logMAR) and BCVA at 1,3, 6, and 12 months postoperatively were 0.39 ± 0.64, 0.02 ± 0.19, -0.03 ± 0.16, -0.05 ± 0.14, and -0.08 ± 0.12, respectively (p<0.01). BCVA (logMAR) at 1, 3, 6, and 12 months postoperatively significantly improved compared with preoperative BCVA (P < 0.01) (Fig 3B). In Group A, preoperative BCVA (logMAR) compared to BCVA at 1,3, 6, and 12 months postoperatively were 0.01 ± 0.35, -0.07 ± 0.13, -0.13 ± 0.07, -0.12 ± 0.09 and -0.12 ± 0.08, respectively (p = 0.066); -0.02 ± 0.14, -0.07 ± 0.10, -0.07 ± 0.13, -0.09 ± 0.10 and -0.11 ± 0.10, respectively (p = 0.106) in Group B; and 0.92 ± 0.61, 0.15 ± 0.21, 0.07 ± 0.16, 0.03 ± 0.16 and -0.02 ± 0.13, respectively (p<0.01) in Group C. BCVA (logMAR) at 1, 3, 6, and 12 months postoperatively significantly improved compared with preoperative BCVA in Group C (P < 0.01) (Fig 3D), but did not change throughout the follow-up period in Groups A and B (Fig 3D).

## Discussion

There are numerous reports describing the changes in visual acuity after PPV in RRD [1–4]. However, there is scant research on the changes in postoperative metamorphopsia over time, especially in PPV. Murakami et al., in their 12-month follow-up study, reported that postoperative metamorphopsia improved with time but tended to persist, and was significantly worse in patients with preoperative foveal detachment after 23-gauge (23G) or 25-gauge (25G) PPV or scleral buckling procedure [8]. Consistent with their report, we found that postoperative metamorphopsia improved with time but persisted during the 12-month follow-up period in all patients, and was significantly more severe in patients with preoperative foveal detachment after 27GPPV. Previous studies have reported a strong relationship between preoperative foveal detachment and postoperative metamorphopsia, occurring in 66.7% to 88.6% of RRD cases involving foveal detachment after scleral buckling procedure [12] and after PPV with/without encircling procedure or scleral buckling procedure [13]. Our study has shown similar results, with postoperative metamorphopsia occurring in 23/33 eyes (69.7%) of RRD cases involving foveal detachment even after 27GPPV. After PPV for RRD involving the fovea, frequent occurrence of downward displacement of the reattached retina and the possibility of strong link between postoperative metamorphopsia and this retinal displacement has been reported [14, 15]. These findings suggest that once foveal detachment has occurred, postoperative metamorphopsia may be unavoidable regardless of the type of surgical procedures.

On the other hand, there are studies which have reported that postoperative metamorphopsia can occur in RRD without preoperative foveal detachment after 23GPPV, 25GPPV, or scleral buckling procedure [5] and PPV or scleral buckling procedure [10]; however, the details are unknown. Therefore, we investigated the relationship between the proximity of RRD to the fovea and postoperative metamorphopsia after 27GPPV. Using multiple regression analysis, we found a significant association between preoperative RRD involving the fovea, graded by the proximity of the RRD to the fovea that was assessed on the optical coherence tomography (OCT) images, and the onset of postoperative metamorphopsia. Zhou et al., in an analysis of 380 eyes, found that among 126 eyes without preoperative foveal detachment, metamorphopsia occurred in 33 eyes, along with ERM, macular hole (MH), and subretinal fluid (SRF) in 3 eyes, 2 eyes, and 1 eye, respectively, after PPV or scleral buckling procedure. However, they reported that postoperative OCT findings were normal in 27 eyes [10]. Okamoto et al. also reported in their investigation of 129 eyes that out of 69 eyes without preoperative foveal detachment, postoperative metamorphopsia occurred in 9 eyes, ERM, MH, and SRF occurred in 4 eyes, 1 eye, and 1 eye, respectively, though postoperative OCT findings were normal in 3 eyes, after 23GPPV, 25GPPV, or scleral buckling procedure [5]. These reports assumed that SRF may temporarily reach the fovea during surgery if the edge of the RRD was close to the fovea and this may cause postoperative metamorphopsia, even in the absence of preoperative foveal detachment. Our results supported the assumptions of these studies, providing evidence for the relationship between the proximity of RRD to the fovea and the risk of postoperative metamorphopsia even after 27GPPV. These results suggest two things: first, there is a risk of unpredictable postoperative metamorphopsia in RRD without preoperative foveal detachment, particularly in RRD that has invaded the vascular arcade regardless of the type of surgical procedures. Second, unlike what is seen in the eyes with preoperative foveal detachment, careful operative maneuvers to avoid the intraoperative SRF inflow to the fovea during surgery may prevent postoperative metamorphopsia in RRD without preoperative foveal detachment. For example, a supplemental therapy with perfluorocarbon or an intentional creation of a drainage site may be able to prevent intraoperative SRF inflow to the fovea and may affect the rates of postoperative metamorphopsia. It should be investigated as further study.

Besides foveal detachment as a preoperative factor, numerous postoperative factors such as the presence of SRF and an abnormality of the outer retinal microstructure, or postoperative retinal morphological abnormalities, such as macular edema, MH, and ERM can contribute to the occurrence of postoperative metamorphopsia [5, 8, 10]. In our multiple regression analysis, we found correlations between postoperative metamorphopsia and preoperative foveal detachment, and the disruption of EZ, a postoperative factor. A previous study with multivariate analysis has suggested that preoperative foveal detachment, postoperatively disrupted external limiting membrane junction, and postoperative SRF significantly correlated with postoperative metamorphopsia [10]. Murakami et al. also report a significant association between postoperative metamorphopsia and preoperative foveal detachment and postoperative disruption of the interdigitation zone [8]. These results suggest that postoperative abnormalities in the outer retinal microstructure of the fovea are a strong predictor of postoperative metamorphopsia, along with preoperative foveal detachment. Moreover, the abnormality of the postoperative outer retinal microstructure was significantly more common in RRD with preoperative foveal detachment in our study. This suggests that the potential abnormality and dysfunction of the outer retinal microstructure has occurred by the time RRD involves the fovea. It may be important to consider different strategies in treating RRD without preoperative foveal detachment, particularly when it is already invading the vascular arcade, to avoid transient foveal detachment.

In our investigation, the development of ERM was observed postoperatively in 13/77 (16.9%) eyes, but there was no correlation between the presence of ERM and postoperative metamorphopsia. Murakami et al., in their study of 47 eyes, found that although ERM occurred postoperatively in 11 eyes (23.4%), there was no correlation between its occurrence and the severity of postoperative metamorphopsia [8]. It is reported that, in general, 80–85% of patients with idiopathic ERM complain of metamorphopsia [16, 17]. The lack of correlation between the presence of ERM and postoperative metamorphopsia may suggest the greater influence of factors other than ERM on the occurrence of metamorphopsia after surgery for RRD. We believe that the extent of foveal involvement is the main factor.

There are several limitations to this study. This was a retrospective study, and the sample size was relatively small. Our results need to be validated through future prospective studies with a larger sample size. However, considering postoperative visual function in a multi-faceted manner, it is important to assess not only the visual acuity but also postoperative metamorphopsia. We believe the following findings are significant: preoperative foveal involvement of the RRD was found to be associated with postoperative metamorphopsia, and in RRD without preoperative foveal detachment invading the vascular arcade, there is a risk of unforeseen postoperative metamorphopsia.

To conclude, postoperative metamorphopsia after 27GPPV for RRD was associated with varying degrees of preoperative foveal RRD and postoperative abnormality of the subfoveal outer retinal microstructure. Furthermore, meticulous surgical maneuvers are required for RRD without preoperative foveal detachment that has invaded the vascular arcade to avoid the intraoperative SRF inflow to the fovea during surgery, because it can occasionally lead to unforeseen postoperative metamorphopsia.

## Supporting information

S1 Data (XLSX)
